# Supplementary material for: HiSNAP trial—a multicentre, randomised, open-label, blinded end point, safety and efficacy trial of conventional (300 mg/kg) versus higher doses of acetylcysteine (450 mg/kg and 600 mg/kg) in patients with paracetamol overdose in the UK: study protocol
Source: BMJ Open. 2025 Mar 22;15(3):e097432. doi: 10.1136/bmjopen-2024-097432 (PMC11977480; doi:10.1136/bmjopen-2024-097432)
Supplement: online supplemental file 1 [file bmjopen-15-3-s001.pdf]

Patient Information Sheet

A trial of conventional versus higher dose acetylcysteine in patients with paracetamol overdose (HiSNAP Trial)

You are invited to take part in a research study. We appreciate this may be a difficult time for you. To help you decide whether or not to take part, it is important for you to understand why the research is being done and what it will involve. Please take time to read the following information carefully. Talk to others about the study if you wish. One of our team will go through the information sheet with you and answer any questions that you may have. Please ask us if there is anything that is not clear or if you would like more information and/or more time to decide whether or not you wish to take part.

What is the purpose of the study?

As you may know, paracetamol can be harmful to the liver when too much has been taken. To help prevent liver damage a medicine known as acetylcysteine (NAC) is given. Despite having this medicine, 1 in 10 patients go on to develop some liver damage. NAC can also cause side-effects such as allergic reactions that can be unpleasant for the patient. In this area there is a lack of knowledge to help the medical staff improve patient care.

It is unknown whether or not giving higher doses of NAC is a better way of preventing liver damage. This trial will determine whether increasing the dose of NAC results in an increase in the breakdown of paracetamol, without causing too many harmful or unacceptable side-effects.

We will ask 90 patients in Scotland to take part in the study. .

Why have I been invited to take part?

We are inviting you to take part in this study because you have taken too much paracetamol and may need treatment to stop this paracetamol from damaging your liver.

Do I have to take part?

No, it is up to you to decide whether or not to take part. If you do decide to take part, you will be given this information sheet to keep and be asked to sign a consent form. You are still free to withdraw at any time and without giving a reason. Deciding not to take part or withdrawing from the study will not affect the healthcare that you receive, or your legal rights.

What will happen if I take part?

A member of the research team will speak to you to discuss your participation in this study and to make sure you understand everything. We will give you time to decide if you want to take part. At most this could be up to an hour but may be only 10-15 minutes if you feel happy to make a decision. This is to make sure there is no delay to your treatment. You will then be asked to give written consent by signing a consent form.

We will ask you to take a urine pregnancy test if there's a chance you could be pregnant as we don't know whether the higher doses are safe for unborn babies so we would not include you if you are. If you are breastfeeding, you should also not take part. You should avoid getting pregnant until the study medication has left your body. This will be 24 hours after the infusion finishes. If you were to have sex in this time, you should use a highly effective method of contraception to avoid getting pregnant. This includes any hormonal contraception that stops you ovulating (pill, injection, implant) or the coil. If you or your partner have had an operation to be sterilised (having your 'tubes tied/vasectomy), this is also effective.

If you become pregnant in the 7 days after you have joined the study, we would review your medical records until the end of the pregnancy to look at the baby's health.

The medical team treating you will look at the paracetamol you have taken, when you took it and the amount of paracetamol in your blood. They will then decide whether you need treatment and we can confirm if you are suitable to join the study.

If you take part in the study, you will be put at random (like tossing a coin) into one of three treatment groups:

1. Standard NAC dose given through a drip over 12 hours as per the normal hospital treatment. This dose is 300 mg per kilogram of your weight.

OR

2. 50% more of the NAC treatment given through a drip over 12 hours. This dose is 450 mg per kilogram of your weight.

OR

3. Double the standard NAC treatment given through a drip over 12 hours. This dose is 600 mg per kilogram of your weight.

You will stay in hospital during the course of your treatment. As part of your normal care a cannula (small plastic tube) will be inserted in a vein in your forearm to receive the NAC. NAC is given as 2 drip bags and this treatment will take 12 hours. The only difference is you may be given a higher dose.

Before and after your treatment you will be asked about any side-effects you are experiencing from the paracetamol overdose and the NAC on a very short questionnaire. If you leave hospital before we are able to give you this questionnaire, we will phone you to ask how you felt.

Blood samples will be collected as part of your normal care for the medical staff to review and we will look at the results of these. We will take an extra blood sample for the research study before your treatment starts. When your treatment finishes, the medical team will be taking a blood sample for your normal care and we will ask for a little extra to be collected for research. The most we will take at any one time will be 30 ml (about 2 tablespoons full). If we are unable to take an extra sample for research, we will ask the hospital lab to use any left-over blood they have once they have completed their tests.

Your urine will be collected in a container for the duration of your drip infusion (12 hours) to look at how the paracetamol is being broken down in your body.

Taking part in the study will not mean you spend longer in hospital than you would normally when receiving this treatment and you will not have to come back to hospital as part of this study. One week after you receive the NAC treatment the trial team will check your medical records to see if you fully recovered.

We will write to your GP to let them know you have taken part in the study.

If you were to lose capacity to make decisions for yourself after you join the study, we would continue to follow you up until the end of the study using your medical notes and keep any samples and data we have collected.

If any new information becomes available about the safety of this treatment which we needed to let you know about, we would contact you using the contact information stored in your medical records.

There may be more than one research study that you are suitable to take part in. If this is the case, the research team will give you the information to consider.

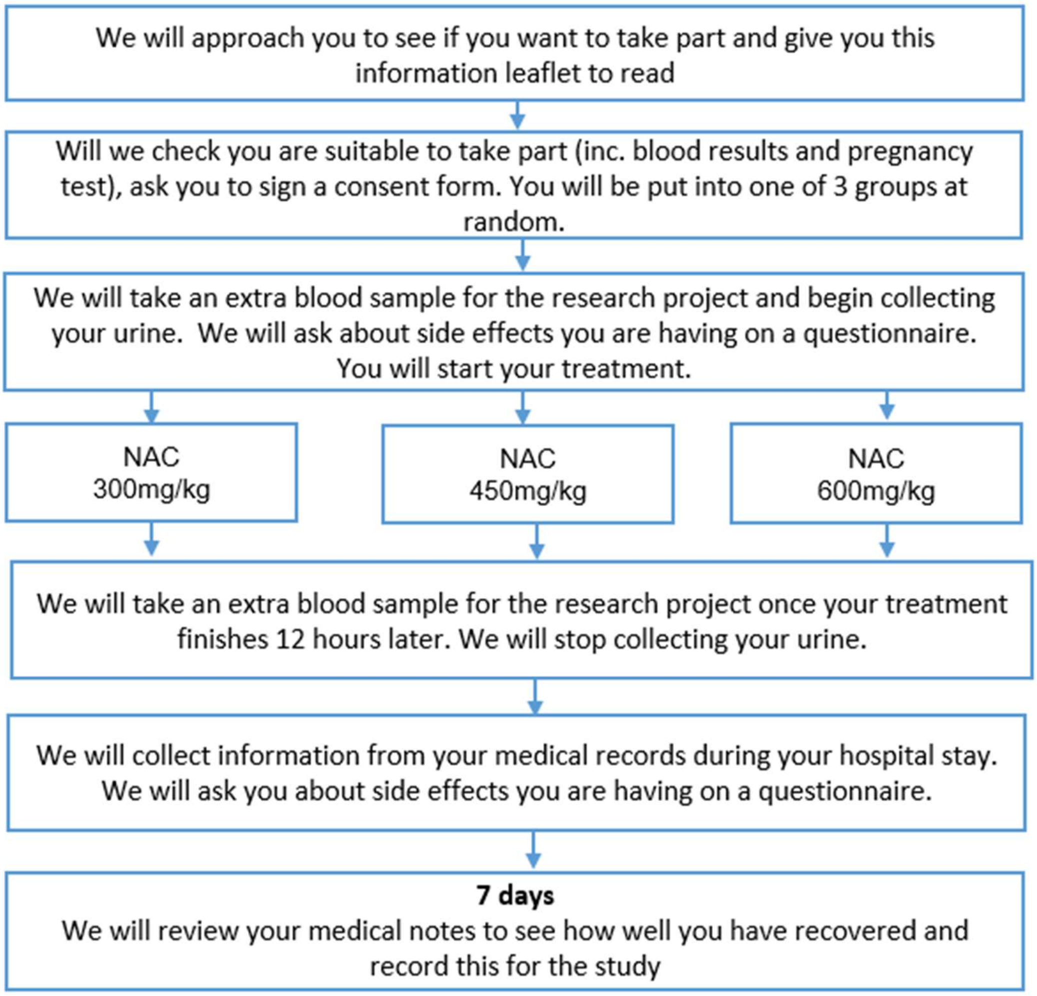

What are the possible benefits of taking part?

There are no direct benefits to you taking part in this study, but the results from this study might help to improve the healthcare of patients in the future.

What are the possible disadvantages of taking part?

Patients treated with NAC may experience side effects, the most common being nausea, vomiting, feeling flushed and skin rash (affects between 1 in 100 and 1 in 10 patients).

Less commonly, patients have more side effects such as changes to their blood pressure, irregular heart rhythm, feeling wheezy, or swelling of the tongue or lips (affects between 1 in 1,000 and 1 in 100 patients). These side effects may be more likely to occur if you are in one of the groups who receive a higher dose of NAC. In most cases, the symptoms subside after stopping the medicine temporarily. When the symptoms are troublesome, the medical team can give you other treatments that may help. We see these reactions in around 1 in 50 patients who have this treatment.

The need for two additional blood samples may cause some minor discomfort.

What if there are any problems?

If you have a concern about any aspect of this study please contact a member of the research team who will do their best to answer your questions (in person or by phoning <INSERT PHONE NUMBER>).

In the unlikely event that something goes wrong and you are harmed during the research due to someone's negligence then you may have grounds for a legal action for compensation against your local NHS Trust but you may have to pay your legal costs. The normal National Health Service complaints mechanisms will still be available to you (if appropriate).

What will happen if I don't want to carry on with the study?

You are free to withdraw at any time and without giving a reason. Deciding not to take part or withdrawing from the study will not affect the healthcare that you receive, or your legal rights. If you withdraw from the study, the information which has been collected about you whilst you have been in the study will be used as part of the results of the research.
If you withdraw from the study, you would continue to receive the NAC which is the normal treatment so that you receive the total dose of medicine required.

If you chose to stop the study, we will ask you whether you want to:

1. Stop higher doses of NAC (if you are being given this) but continue to let us collect blood samples and new information about you from you and your medical records.

2. Stop higher doses of NAC (if you are being given this) and stop extra blood and urine samples but continue to let us collect new information about you. We will keep the information we have already collected to that point, including the blood and urine samples we have taken.
3. Stop all participation in the study including collection of new information about you. We will keep the information we have already collected to that point, including the blood and urine samples we have taken.

What happens when the study is finished?

When you complete the 12 hours of treatment, the medical team will decide, on the basis of your blood results, whether you need any further treatment with NAC. If you do, you will receive the standard dose of NAC given routinely given at the hospital. If your blood results are satisfactory, you will not require more treatment.

At the end of the research, the paper records will be stored for 5 years. After this period, they will be disposed of securely. Personal data collected for the study will be kept at the research site for a maximum of one year after the end of the research.

The electronic data will be held indefinitely on a secure server by the University of Edinburgh.

We will make the study data available for other researchers to look at so the knowledge gained from this research will be as useful as possible, this may include researchers from outside the UK / European Economic Area. Before we make it available, we will make sure it does not contain any data which could be used to identify you.

Will my taking part be kept confidential?

All the information we collect during the course of the research will be kept confidential and there are strict laws which safeguard your privacy at every stage.

How will we use information about you?

We will need to use information from you and your medical records for this research project.

We will collect your Community Health Index (CHI) number or NHS number. Note that the CHI is a population register, used in Scotland for health care purposes. The CHI number uniquely identifies a person on the index and is personal identifiable information. Your CHI number or NHS number is being collected to allow us to link to your medical records and in case we need to contact you again. This information will be kept within your local NHS Trust. .

Other personal identifiable information collected will include your name, date of birth, sex at birth, gender and ethnicity and phone number. People will use this information to do the research or to check your records to make sure that the research is being done properly.
People who do not need to know who you are will not be able to see your personal information. Your data will have a code number assigned instead.

We will keep all information about you safe and secure in a database held by the Edinburgh Clinical Trials Unit at the University of Edinburgh or on computers at your local NHS Trust.

Once we have finished the study, we will keep some of the data so we can check the results. We will write our reports in a way that no-one can work out that you took part in the study.

What are your choices about how your information is used?

You can stop being part of the study at any time, without giving a reason, but we will keep information about you that we already have.

If you choose to stop taking part in the study, we would like to continue collecting information about your health from your electronic medical records. If you do not want this to happen, tell us and we will stop.

We need to manage your records in specific ways for the research to be reliable. This means that we won’t be able to let you see or change the data we hold about you.

Where can you find out more about how your information is used?

You can find out more about how we use your information

- at www.hra.nhs.uk/information-about-patients/
- our leaflet available from **www.hra.nhs.uk/patientdataandresearch**
- by asking one of the research team
- by sending an email to the Data Protection Officer at University of Edinburgh (dpo@ed.ac.uk) or NHS Lothian (Loth.DPO@nhslothian.scot.nhs.uk)
- by ringing us on 0131 242 3863.

What will happen to the results of the study?

The results will be published in a relevant medical journal and presented to other medical staff at conferences. You will not be identified in any report or publication.
Once the study has been published a summary of the findings will be available on the Edinburgh Clinical Trials website https://www.ed.ac.uk/usher/edinburgh-clinical-trials/our-studies/all-current-studies/hisnap.

Who is organising and funding the research?

This study has been organised by the Edinburgh Clinical Trials Unit and Sponsored by the University of Edinburgh and NHS Lothian. The study is being funded by the Chief Scientist Office.

Who has reviewed the study?

The study proposal has been reviewed by experienced researchers at the University of Edinburgh and in the NHS. All research in the NHS is also looked at by an independent group of people, called a Research Ethics Committee. A favourable ethical opinion has been obtained from East Midlands - Derby Research Ethics Committee. Medicines and Healthcare products Regulatory Agency (MHRA) and NHS management approval have also been obtained.

Researcher Contact Details

If you have any further questions about the study, please contact <<Local PI/Research team contact >>

Independent Contact Details

If you would like to discuss this study with someone independent of the study, please contact Dr Neeraj Dhaun, Bean.Dhaun@ed.ac.uk.

Complaints

If you wish to make a complaint about the study please contact: <<INSERT LOCAL COMPLAINTS INFO>>

Participant ID: ☐☐☐☐-☐☐

CONSENT FORM HiSNAP

Please **initial** box

1. I confirm that I have read and understand the information sheet for the above study. ☐

*Date (DD MMM YYYY)	*Version Number

**complete during consent process*

2. I have had the opportunity to consider the information, ask questions and have had these questions answered satisfactorily. ☐
3. I understand that my participation is voluntary and that I am free to withdraw at any time, without giving any reason and without my medical care and/or legal rights being affected. ☐
4. I give permission for the research team to access my medical records for the purposes of this research study. ☐
5. I understand that relevant sections of my medical notes and data collected during the study may be looked at by individuals from the Sponsor (University of Edinburgh and/or NHS Lothian), from regulatory authorities or from the NHS organisation where it is relevant to my taking part in this research. I give permission for these individuals to have access to my data and/or medical records. ☐
6. I give permission for my personal information (including name, date of birth, sex at birth, gender, ethnicity, phone number and consent form) to be retained on NHS servers and for my ethnicity, gender, sex and age to be passed to the University of Edinburgh / Edinburgh Clinical Trials Unit for administration of the study. ☐
7. I give permission for my Community Health Index (CHI) number to be collected and retained on NHS servers. ☐
8. I give permission for the research team to continue to follow me up and keep any samples and data already collected if I were to lose capacity to make decisions for myself after I join the study. ☐
9. I give permission for the research team to keep any samples and data already collected if I decide to withdraw from the study. ☐
10. I agree to my General Practitioner being informed of my participation in the study. ☐
11. I understand that data collected about me during the study may be converted to anonymised data. ☐
12. I agree to my anonymised data being used for future ethically approved studies. Yes ☐ No ☐
13. I agree to take part in the above study. ☐

Name of Person Giving Consent	Date	Signature
Name of Person Receiving Consent	Date	Signature

1x original – into Site File; 1x copy – to Participant; 1x copy – into medical record
