# Supplementary material for: HiSNAP trial—a multicentre, randomised, open-label, blinded end point, safety and efficacy trial of conventional (300 mg/kg) versus higher doses of acetylcysteine (450 mg/kg and 600 mg/kg) in patients with paracetamol overdose in the UK: study protocol
Source: BMJ Open. 2025 Mar 22;15(3):e097432. doi: 10.1136/bmjopen-2024-097432 (PMC11977480; doi:10.1136/bmjopen-2024-097432)
Supplement: Supplementary file 3 [file bmjopen-2024-097432.reviewer_comments.pdf]

PEER REVIEW HISTORY

BMJ Open publishes all reviews undertaken for accepted manuscripts. Reviewers are asked to complete a checklist review form and are provided with free text boxes to elaborate on their assessment. These free text comments are reproduced below.

ARTICLE DETAILS

Title (Provisional)

Study protocol: HiSNAP trial - a multi-centre, randomised, open label, blinded end-point, safety and efficacy trial of conventional (300mg/kg) versus higher doses of acetylcysteine (450mg/kg and 600mg/kg) in patients with paracetamol overdose in the United Kingdom

Authors

Humphries, Christopher; Clarke, Ellise; Eddleston, Michael; Gillings, Marianne; Irvine, Sian; Keating, Liza; Miell, Anna; Milne, Lynsey; Muir, Laura; O'Brien, Rachel; Oatey, Katherine; Raman, Rajendra; Thanacoody, Ruben; Tuck, Sharon; Weir, Christopher; Wood, David; Dear, James W; HiSNAP Trial Investigators, HiSNAP Trial Investigators

---

This article was not externally reviewed at BMJ Open. Protocols that have been independently assessed prior to submission to BMJ Open are usually fast-tracked to publication on the grounds that further substantial changes will not be possible. This independent assessment will usually be external, independent review for both a substantial grant award from a non-commercial or government funder and ethics approval.
